# Extramedullary Plasmacytoma of the Oral Cavity: A Rare Case in the Buccal Mucosa

**DOI:** 10.1002/ccr3.9612

**Published:** 2025-01-09

**Authors:** Armin Khaleghi, Nafise Shamloo

**Affiliations:** ^1^ Dental Research Center, Research Institute of Dental Sciences, School of Dentistry Shahid Beheshti University of Medical Sciences Tehran Iran; ^2^ Department of Oral and Maxillofacial Pathology, School of Dentistry Shahid Beheshti University of Medical Sciences Tehran Iran

**Keywords:** buccal mucosa, extramedullary plasmacytoma, multiple myeloma, oral cavity

## Abstract

Extramedullary plasmacytomas (EMP) are uncommon solitary tumors composed of neoplastic plasma cells occurring outside the bone. These lesions are of clinical significance due to their potential progression to multiple myeloma (MM), a more systemic and serious plasma cell malignancy. Although EMPs primarily arise in the head and neck region, cases involving the oral cavity are particularly rare. This report presents a case of EMP in a 59‐year‐old Iranian female, who developed a mass on the left buccal mucosa, an atypical site for this type of lesion. The patient presented with a 1‐month history of a painful swelling on her left buccal mucosa. Clinical examination revealed a firm, exophytic lesion with a smooth surface. Histopathological analysis, supported by immunohistochemical staining, confirmed the diagnosis of EMP. The lesion was treated successfully through a combination of surgical excision and radiotherapy at a dose of 40 Gy. This case underscores the importance of early diagnosis and treatment of EMP, particularly in rare locations, to prevent progression to MM and improve patient outcomes. Our findings contribute to the limited body of literature on EMPs in the oral cavity, highlighting the need for awareness of such presentations among clinicians.

**Summary**

- Extramedullary plasmacytoma can present in atypical locations, such as the buccal mucosa.
- Clinicians should consider EMP in the differential diagnosis of oral soft tissue lesions to enable early diagnosis and prevent progression to multiple myeloma.

## 1 Introduction

Plasmacytoma is characterized by a mass of neoplastic monoclonal plasma cells in either bone which is called solitary bone plasmacytoma (SBP) or in soft tissue, called EMP. SBP is defined as a single lytic lesion with or without soft tissue extension, whereas EMP is a soft tissue lesion that is not in contact with the bone [1]. SBP mostly occurs in the bones of the axial skeleton, such as the vertebra and skull [2]. On the contrary, approximately 25% of EMPs develop in the head and neck region [2], and most frequently in the nasal cavity and nasopharynx [3, 4, 5]. The average age of the patients with EMP is 55 years [2]. The incidence rate rises with advancing age; however, it is less prominent at older ages in comparison with MM [6, 7].

Understanding the distinctions between different types of plasmacytomas is vital for clinicians, especially when diagnosing and treating cases in unusual location like the oral cavity. Our report presents an EMP in the buccal mucosa, which highlights the importance of awareness and careful identification of such presentations.

## 2 Case History/Examination

A 59‐year‐old healthy female referred to Shahid Beheshti Dental School with a complaint of swelling on her left buccal mucosa. The patient had noticed the lesion approximately 1 month ago, with pain being the only associated symptom. Clinical examination showed a firm exophytic lesion with a smooth surface on the left buccal mucosa, measuring 5 × 3 × 1 cm. Radiographs from the jaws and chest showed no sign of bone involvement, which ruled out multiple myeloma.

## 3 Methods

**FIGURE 1:**
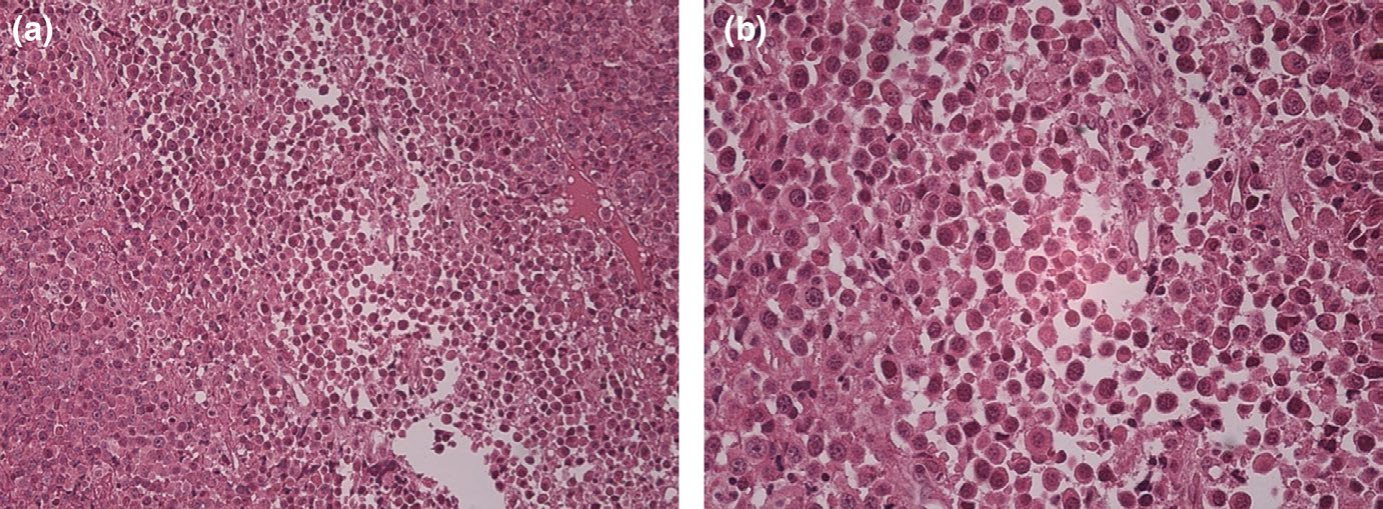
Sheets of malignant plasma cells with eccentric nuclei with a magnification of (a) x10 and (b) x20.

Lymphoma, leukemia, and undifferentiated carcinoma were suggested as the differential diagnosis for this lesion. An incisional biopsy was performed and the specimen was sent to the pathology department. Microscopic views of the lesion are illustrated in Figure 1. Histopathologic examination showed diffuse sheets of neoplastic plasma cells with various degrees of differentiation including binucleated and multinucleated forms. Chronic inflammatory cell infiltration, Russel bodies, histiocytes, and a few mitoses were seen. The lesion was covered by ulcerated parakeratinized stratified squamous epithelium, which was replaced with fibrinopurulent membrane. Bone invasion was not evident.

**FIGURE 2:**
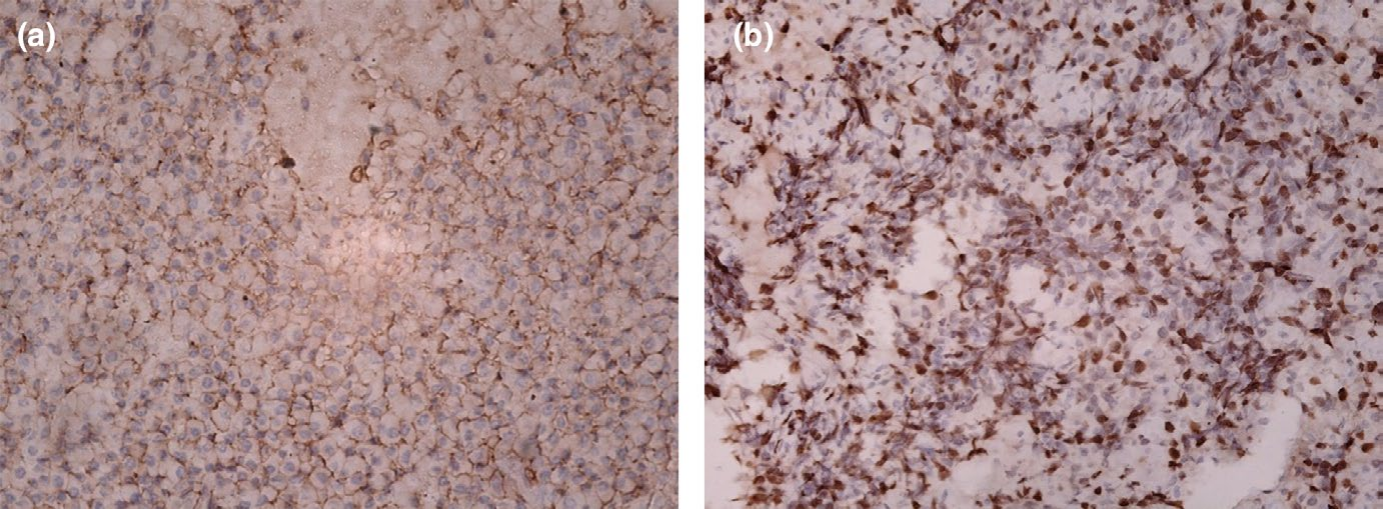
Positive immunohistochemical reaction of the lesional cells for (a) CD138 and (b) Ki67, with a magnification of x20.

**FIGURE 3:**
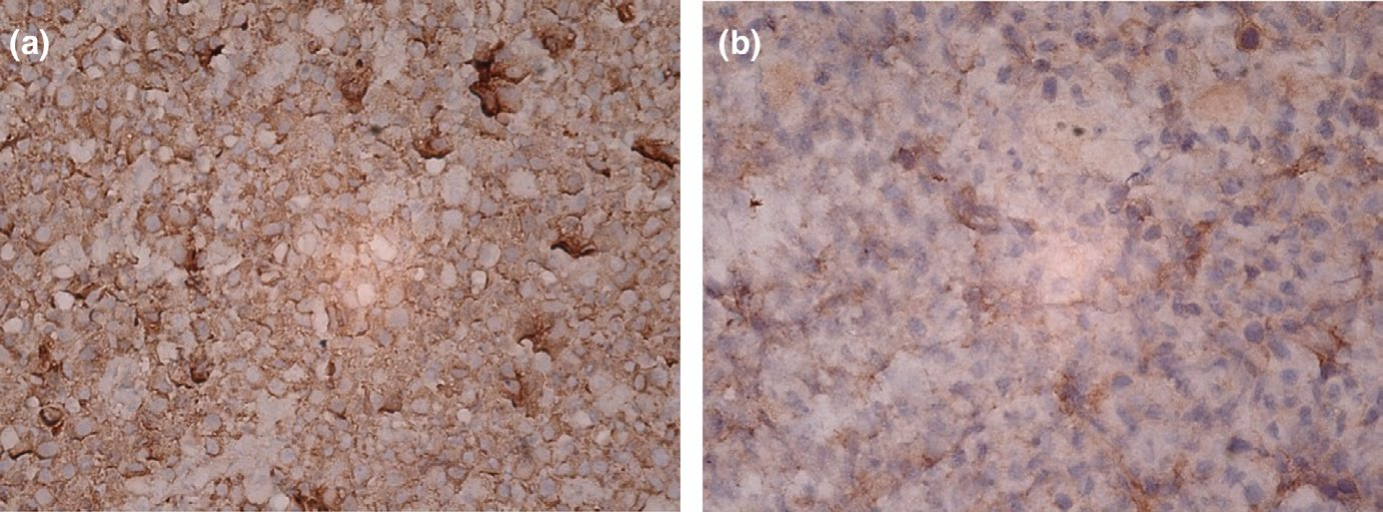
Immunohistochemical study shows (a) a uniform reaction for lambda in most plasma cells, and (b) positivity for kappa in a few plasma cells, with a magnification of x40.

Immunohistochemistry (IHC) staining for Ki67, CD3, CD20, CD138, HMB‐45, Melan A, and vimentin was done. Ki67 index was more than 50% and CD138 was also positive. All other IHC markers were negative. Lambda light chain restriction was positive in most plasma cells and kappa light chain restriction was positive in a few. Figures 2 and 3 show the photographs taken from IHC stating.

## 4 Conclusion and Results

Based on the histopathologic examination and IHC, EMP was considered as the diagnosis. The tumor was locally excised and radiotherapy was done with a dose of 40 Gy. Three‐month follow‐up showed no signs of recurrence.

## 5 Discussion

EMPs typically present as well‐circumscribed, non‐tender soft tissue masses, with no systemic evidence of MM [2]. Although EMPs commonly involve the nasopharynx and paranasal sinuses [8], our case presents an EMP on the buccal mucosa, a less common site. Gingiva, labial mucosa, tongue, uvula, and soft palate are the other sites in the oral cavity in which EMP was reported [9]. In our case, EMP occurred in a 59‐year‐old woman, although there is a slightly stronger male predilection, approaching a 3:1 male‐to‐female ratio [2]. Symptoms of most EMPs are not typical. Nasal obstruction, epistaxis, facial pain, or hoarse voice commonly occur in head and neck EMPs [10].

The incidence of EMP is lower than SBP and accounts for only about 3% of all plasma cell neoplasia [8]. EMP seems to have a better prognosis compared with SBP. The relationship between EMP and MM is not clear nowadays. About one‐third of the patients with EMP are diagnosed as MM within 2 years after the initial symptoms [11]. Some studies showed EMP may occur in the early MM and the patient could be easily misdiagnosed in this phase [10].

EMP can be confused with other exophytic lesions of the oral cavity, including benign conditions such as fibroma and pyogenic granuloma, or malignant ones such as squamous cell carcinoma, lymphoma, or melanoma [12]. Histological evaluation and IHC confirmation of monoclonality is the preferred diagnostic method. Monoclonality is indicated by the expression of CD138 and/or CD38, and confirmed through Kappa/Lambda light chain restriction or polymerase chain reaction (PCR) methods [1]. In this case, negative results for CD20 and CD3 excluded lymphoma, whereas melanoma was ruled out by negative results of Melan A and HMB‐45. Positive results for the kappa light chain and lambda confirmed the EMP diagnosis.

Diagnostic adjuncts such as fine‐needle aspiration cytology, CT scan, MRI, or PET‐CT can be helpful [10]. For oral lesions, an incisional biopsy is typically sufficient, but for lesions on the gingiva and palate, a CT scan can help determine whether there is bony involvement. Also, MRI or PET‐CT can be used to evaluate the extent of soft tissue involvement when biopsy access is challenging [9].

Plasmacytomas are radiosensitive tumors. The treatment involves local lesion excision with adjunctive radiotherapy, especially when surgical margins are involved. Previous research suggests an optimal dose of 46 Gy to minimize toxicity [13]. The long‐term disease‐free survival rate following radiotherapy is 65% for patients with EMP compared with 30% for those with SBP [14].

This case underscores the importance of considering EMP in the differential diagnosis of atypical oral lesions. Although current diagnostic and treatment strategies are effective, further research is needed to better understand the relationship between EMP and MM, and to optimize management approaches for these conditions.

## Author Contributions

**Armin Khaleghi:** conceptualization, formal analysis, investigation, project administration, writing – review and editing. **Nafise Shamloo:** investigation, methodology, supervision, validation, writing – original draft.

## Consent

Written informed consent was obtained from the patient to publish this report in accordance with the journal's patient consent policy.

## Conflicts of Interest

The authors declare no conflicts of interest.

## Data Availability

The data that support the findings of this study are available upon request from the corresponding author. The data are not publicly available due to privacy or ethical restrictions.
